# Atractylenolide II Alleviates 5-Fluorouracil-Induced Intestinal Toxicity Through Potentially Targeting the TLR4-Mediated MYD88/NF-κB Signaling Pathway: An Integrated In Silico and In Vitro Study

**DOI:** 10.3390/molecules31213968

**Published:** 2026-11-03

**Authors:** Yangzhi Liu, Ru Jia, Yue Liu, Pengpeng Liu, Huiying Xu, Yue Liu, Yuxin Chen, Jingyu Huan, Haoran Yu, Yang Qu, Qian Cai

**Affiliations:** 1 College of Pharmacy, Liaoning University of Traditional Chinese Medicine, Dalian 116600, China; 13190177482@163.com (Y.L.); 18840966696@163.com (R.J.); pengpengliu0411@163.com (P.L.); 13842057731@163.com (H.X.); 18035356706@163.com (Y.L.); 18007411942@163.com (Y.C.); 19862613412@163.com (J.H.); yuhaoran1027@163.com (H.Y.); 2 College of Pharmacy, Gansu Medical College, Pingliang 744000, China; liuyue996@sina.cn

**Keywords:** atractylenolide II, 5-fluorouracil, intestinal toxicity, TLR4

## Abstract

5-Fluorouracil (5-FU) is a potent compound in anti-tumor chemotherapy; however, its clinical utility is hampered by dose-limiting intestinal toxicity. Atractylenolide II (AT-II), a bioactive sesquiterpene derived from Atractylodis Rhizoma, has reported anti-inflammatory and cytoprotective activities. In this study, we employed an integrated strategy combining chemical characterization, computational pharmacology, and in vitro experiments to investigate the protective effect of AT-II against 5-FU-induced intestinal toxicity and clarify its potential target-associated mechanism. UHPLC-QE-MS was applied to profile the constituents of Atractylodis Rhizoma, with AT-II subjected to subsequent computational and experimental analyses. ADMET evaluation, network pharmacology, molecular docking, 100 ns molecular dynamic (MD) simulation and DFT calculation were performed to predict druggability, candidate targets and molecular interaction features. TLR4 and HIF1A were screened as hub candidates. MD simulations revealed that the AT-II–TLR4 complex exhibited much better conformational stability than AT-II–HIF1A. DFT calculations suggested that the lactone moiety of AT-II exhibits pronounced electron-rich features. The DARTS assay provided evidence for a potential interaction between AT-II and TLR4. In IEC-6 cells, 20 μM AT-II rescued 5-FU-impaired cell viability, decreased the release of pro-inflammatory IL-1β, IL-6 and TNF-α, and suppressed the TLR4/MYD88/NF-κB inflammatory cascade by inhibiting NF-κB p65 phosphorylation. Taken together, these results suggest that AT-II protects against 5-FU-induced intestinal toxicity, probably via modulation of TLR4 and downstream MYD88/NF-κB inflammatory signaling, offering preliminary in vitro evidence for developing AT-II to ameliorate chemotherapy-associated intestinal toxicity.

## 1. Introduction

5-Fluorouracil (5-FU) represents a first-line chemotherapeutic agent widely used in the clinical treatment of multiple gastrointestinal tumors and other solid malignancies [1,2]. Unfortunately, severe adverse effects are reported in patients receiving 5-FU, including intestinal toxicity, which is considered the major side effect of 5-FU administration [3,4]. At the cellular level, 5-FU-induced intestinal damage triggers the release of endogenous damage-associated molecular patterns (DAMPs), which activate inflammatory signaling cascades mediated by pattern recognition receptors [5]. Among these signaling pathways, the TLR4/MYD88/NF-κB pathway serves as the core regulatory cascade driving intestinal inflammatory injury [6,7,8]. Activated TLR4 recruits the downstream adaptor protein MYD88, which in turn promotes the phosphorylation and nuclear translocation of NF-κB p65, and ultimately induces excessive transcription and secretion of pro-inflammatory cytokines including IL-1β, IL-6, and TNF-α [1,9,10]. Sustained inflammatory activation further aggravates intestinal epithelial injury. Currently, no effective drugs are available for preventing this side effect. Therefore, exploring safe and effective natural bioactive compounds to alleviate 5-FU-induced intestinal toxicity has important clinical significance.

Atractylodis Rhizoma is a classic traditional Chinese medicinal herb that has been widely applied for the management of gastrointestinal disorders [11,12]. Available reports suggest that formulas containing Atractylodis Rhizoma can alleviate gastrointestinal adverse reactions triggered by tumor chemotherapy [13,14]. Phytochemical studies have demonstrated that sesquiterpenes represent its principal bioactive constituents, which contribute substantially to its gastrointestinal regulatory and anti-inflammatory effects [11]. Atractylenolide II (AT-II), a characteristic sesquiterpene derived from Atractylodis Rhizoma, exhibits diverse pharmacological activities. Numerous studies have demonstrated that AT-II exerts anti-inflammatory, anti-tumor, antioxidant and cytoprotective effects in multiple cell-based models [15,16,17]. Mechanistically, AT-II modulates several kinase-related and transcription factor-associated signaling cascades to exert its effects. However, whether AT-II can exert protective effects against 5-FU-induced intestinal toxicity and the potential molecular mechanisms underlying its action remain unclear.

Network pharmacology and other in silico strategies have emerged as valuable tools to unravel the multi-target mechanisms underlying the bioactivity of natural products [18,19]. Although computational approaches may yield mechanistic predictions, experimental validation remains essential. The drug affinity-responsive target stability (DARTS) assay is particularly adept at evaluating protein–small-molecule interactions without requiring any structural modifications [20]. This method bridges the gap between in silico predictions and bioassays [21,22].

This study carried out an investigation from the perspectives of chemical characterization, computational prediction, and experimental validation. UHPLC-QE-MS was used to profile the chemical constituents of Atractylodis Rhizoma, and AT-II was selected for subsequent analyses. ADMET drug-likeness prediction, network pharmacology target screening, molecular docking, 100 ns MD simulation, and DFT electronic structure calculation were performed to screen core therapeutic targets. Among the hub targets, including TLR4 and HIF1A, TLR4 was prioritized based on molecular dynamic stability assessment, and molecular interaction features of AT-II were further characterized. The DARTS assay was conducted to provide supportive evidence for a potential interaction between AT-II and TLR4. A 5-FU-induced IEC-6 cell injury model was used to explore the protective effect of AT-II and its regulatory effect on downstream inflammatory signaling pathways.

## 2. Results

### 2.1. Characterization of Chemical Constituents of Atractylodis Rhizoma Using UHPLC-QE-MS

**Figure 1:**
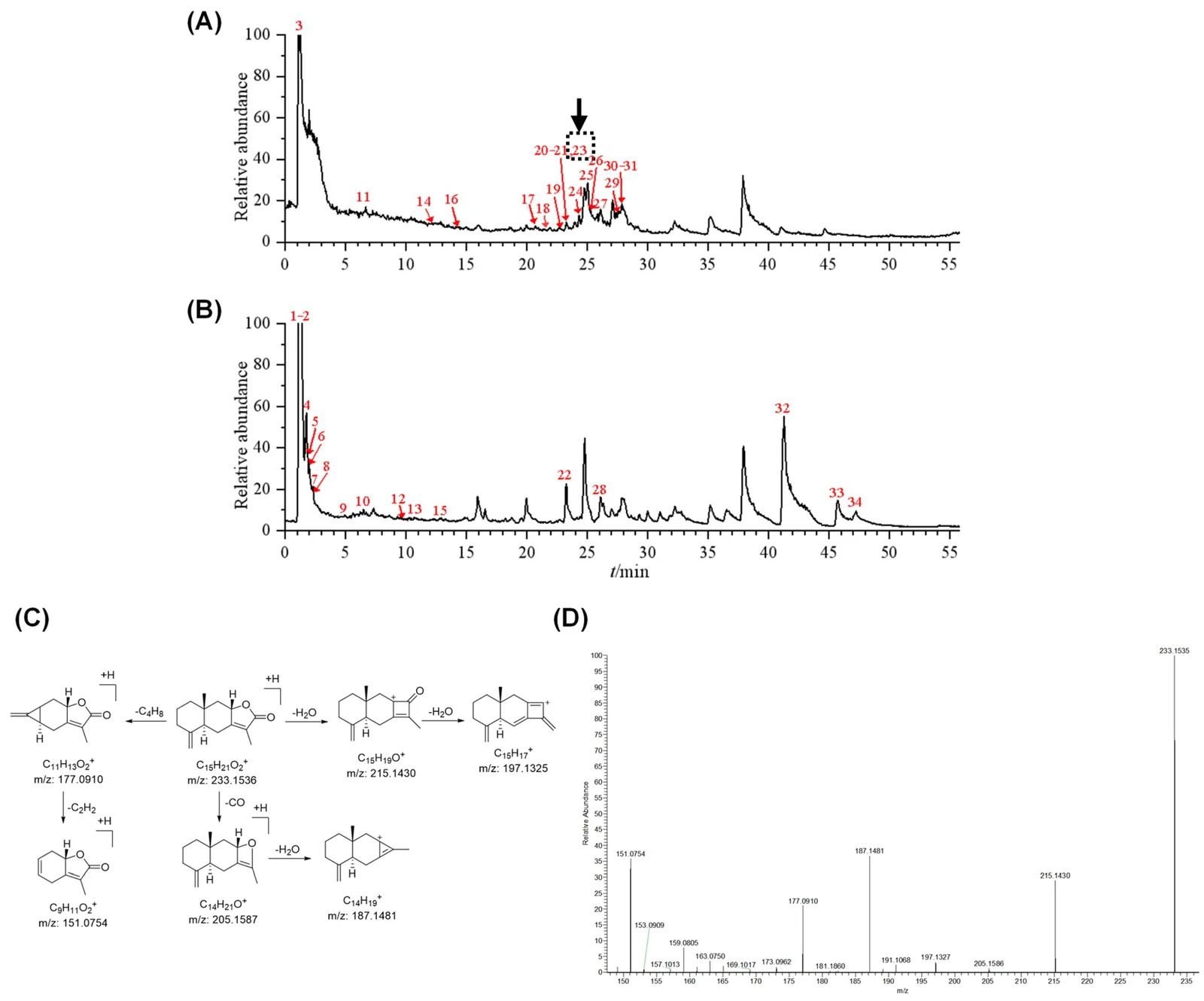
Chemical characterization of AT-II from Atractylodis Rhizoma. (**A**) TIC in positive-ion mode. The peak corresponding to AT-II is marked by the black arrow. (**B**) TIC in negative-ion mode. (**C**) Proposed MS/MS fragmentation pathway of AT-II. (**D**) Representative MS/MS spectrum of AT-II.

The chemical constituents of Atractylodis Rhizoma were profiled using UHPLC-QE-MS. Representative total ion chromatograms are displayed in Figure 1A,B. In total, 34 chemical constituents were tentatively identified based on retention time, accurate molecular mass, and MS/MS fragment characteristics, with detailed compound information summarized in Table S1 and Figure S1. These identified compounds primarily cover sesquiterpenes, organic acids, coumarins and polyacetylenes of Atractylodis Rhizoma. AT-II was selected as the core research candidate for subsequent studies due to its reported anti-inflammatory and cytoprotective bioactivities [15]. The protonated molecular ion of AT-II was detected at m/z 233.1535 [M+H]^+^ with a retention time of 23.32 min, corresponding to the molecular formula C_15_H_20_O_2_ (ppm: −0.46). The diagnostic MS/MS fragment ions arising from successive dehydration and decarbonylation matched previously reported data for AT-II [11]. The deduced MS/MS fragmentation pathway of AT-II is illustrated in Figure 1C,D.

### 2.2. ADMET Properties and Druglikeness Evaluation of AT-II

**Figure 2:**
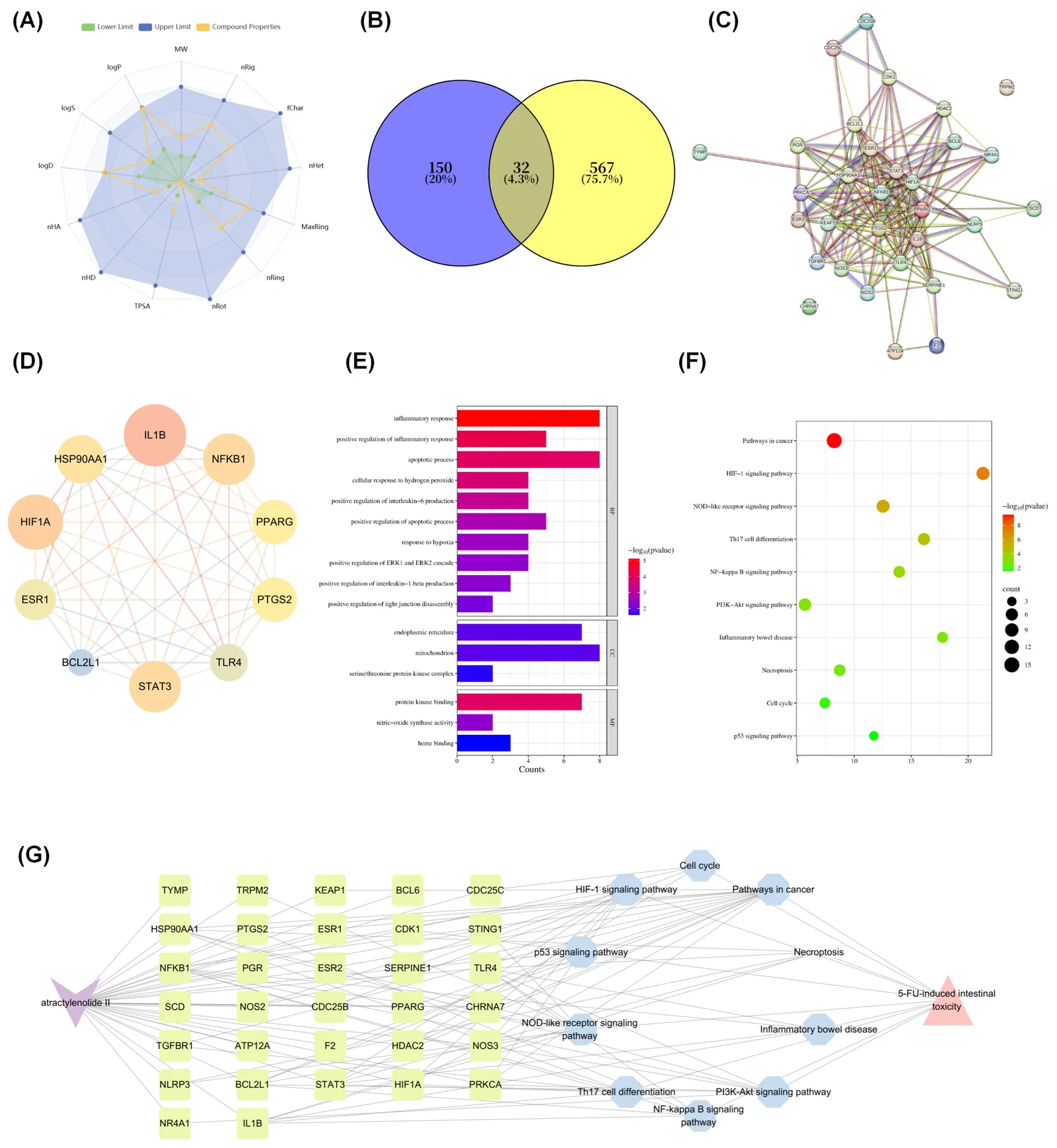
Network pharmacology and druglikeness prediction of AT-II against 5-FU-induced intestinal toxicity. (**A**) Radar chart of ADMET and druglikeness parameters for AT-II. (**B**) Venn diagram showing the intersection of AT-II putative targets and 5-FU-induced intestinal toxicity-related targets. (**C**) Protein–protein interaction (PPI) network of overlapping targets. (**D**) Top 10 hub genes screened by topological analysis. (**E**) GO functional enrichment analysis. (**F**) KEGG pathway enrichment analysis. (**G**) Compound-target-pathway regulatory network of AT-II.

ADMET and safety properties of AT-II were predicted using ADMETlab 3.0. Predicted physicochemical and pharmacokinetic properties together with safety data for AT-II are summarized in Table 1, and the corresponding radar plot is shown in Figure 2A.

AT-II displayed balanced lipophilic–polar features and complied with Lipinski’s rule of five and other standard druglikeness filters.

For intestinal absorption, AT-II displayed promising epithelial permeability. Its Caco-2 permeability value (−4.476) fell above the favorable threshold defined by ADMETlab 3.0, suggesting good epithelial permeation potential in silico. AT-II yielded low probabilities for poor intestinal absorption, insufficient oral bioavailability, and P-gp-mediated efflux, suggesting a low predicted risk of absorption-associated constraints. Since 5-FU-induced intestinal toxicity arises from damage to proliferating intestinal epithelium [23], such predicted intestinal permeability would be a favorable property for candidate compounds in this context.

AT-II is predicted to act as a substrate for multiple CYP450 isoforms, but shows low inhibitory potential toward major CYPs (except CYP2C19), suggesting a low predicted risk of CYP-related drug–drug interactions. Previous studies using human liver microsomes support these predictions for CYP-dependent metabolism [24].

Pharmacokinetic predictions suggest moderate plasma clearance and a short elimination half-life for AT-II, reflecting rapid systemic turnover and no sustained body accumulation. This finding agrees with earlier pharmacokinetic reports describing rapid oral absorption of atractylenolides [25]. Notably, prolonged systemic circulation is not required for local gastrointestinal protective effects.

For safety assessment, AT-II exhibited a low probability (0.026) of hERG-channel blockage, suggesting limited predicted risk of hERG-associated cardiac toxicity. Overall, these in silico predictions suggest that AT-II may possess favorable druglikeness for further research targeting 5-FU-induced intestinal toxicity.

### 2.3. Network Pharmacology Analysis for AT-II Against 5-FU-Induced Intestinal Toxicity

Network pharmacology was used to screen potential therapeutic targets of AT-II for 5-FU-induced intestinal toxicity. After deduplication, 182 valid AT-II targets and 599 disease-related targets for 5-FU-induced intestinal toxicity were retrieved. Venn diagram analysis identified 32 overlapping genes, which were regarded as core drug–disease intersection targets (Figure 2B). These overlapping targets were imported into the STRING database to construct a protein–protein interaction (PPI) network (Figure 2C). Importantly, ten hub genes were identified: *IL1B*, *HIF1A*, *NFKB1*, *STAT3*, *HSP90AA1*, *PPARG*, *PTGS2*, *ESR1*, *TLR4*, and *BCL2L1* (Figure 2D).

GO enrichment analysis was performed to reveal the functional features of these core targets. In total, 226 significantly enriched GO terms were obtained: 162 Biological Process (BP), 48 Molecular Function (MF), and 16 Cellular Component (CC). BP analysis showed prominent enrichment in inflammatory response-related biological processes, such as positive regulation of interleukin-1β production and inflammatory response activation. CC were mainly annotated to cytosol and cytoplasm regions (Figure 2E). KEGG pathway enrichment yielded 74 pathways, and the top ten significantly enriched entries were visualized (Figure 2F). The results suggested that the core targets were mainly enriched in inflammatory bowel disease, the HIF-1 signaling pathway, necroptosis, and insulin signaling pathway, indicating that AT-II may exert protective effects against 5-FU-induced intestinal toxicity by regulating the above inflammatory and stress-related pathways. The compound-target-pathway network further illustrated the multi-target, multi-pathway regulatory features of AT-II (Figure 2G).

### 2.4. Molecular Docking of AT-II with Hub Target Proteins

**Figure 3:**
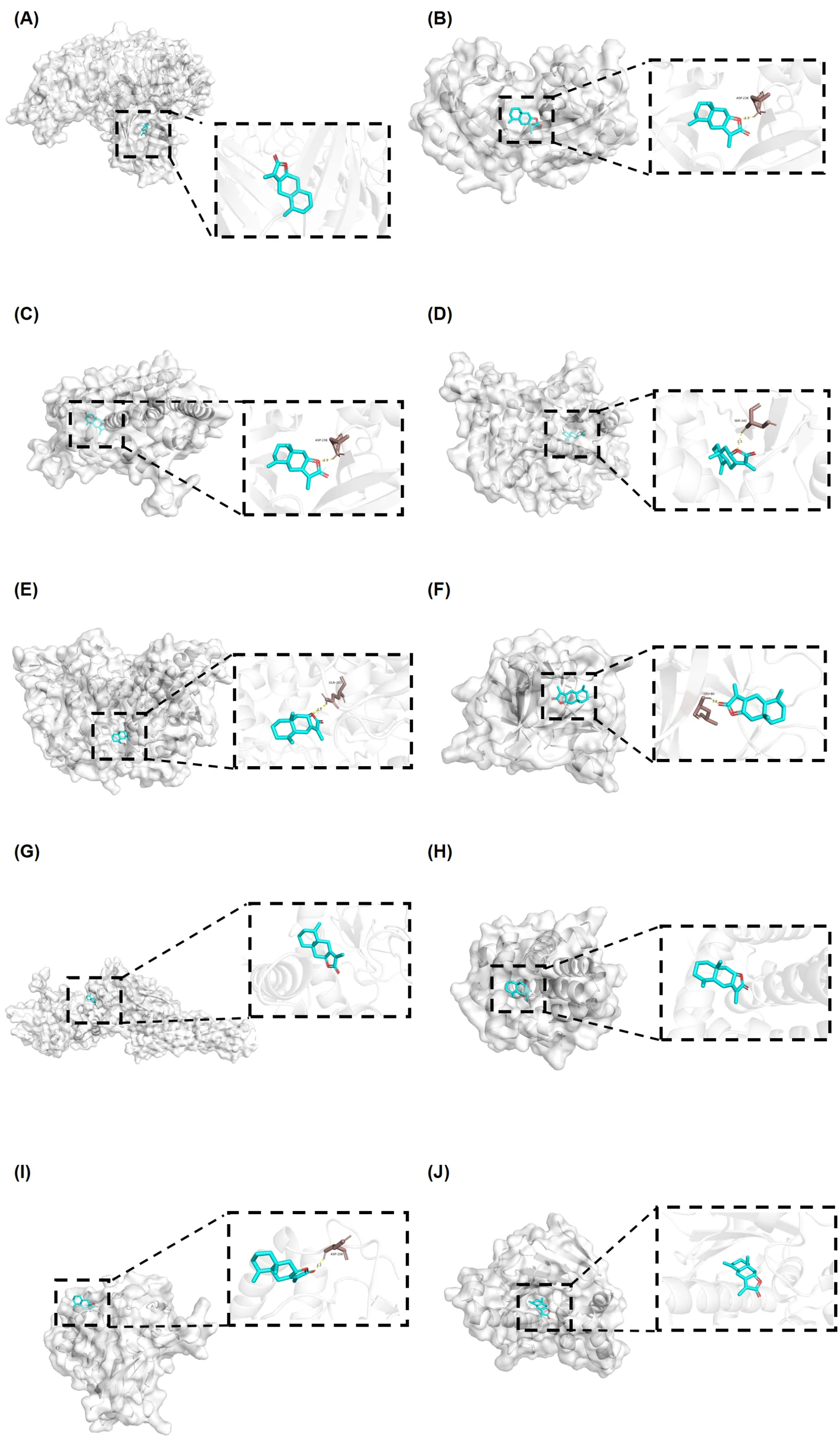
Molecular docking analysis. (**A**–**J**) 3D binding conformations of AT-II complexed with TLR4, HIF1A, ESR1, PPARG, PTGS2, IL1B, STAT3, NFKB1, BCL2L1, and HSP90AA1, respectively.

AT-II and ten hub target proteins (IL1B, HIF1A, NFKB1, STAT3, HSP90AA1, PPARG, PTGS2, ESR1, TLR4, and BCL2L1) identified from the network pharmacology analysis were screened for molecular docking assessment. The resulting binding affinities for AT-II–target complexes are shown in Table 2. The optimal binding poses of ligand–receptor complexes are presented (Figure 3). All hub proteins exhibited favorable binding affinity toward AT-II, with binding energies ranging from −6.1 to −9.1 kcal/mol, suggesting favorable binding interactions between AT-II and these targets. Combined with docking affinity, network topological significance, KEGG enrichment results, and documented core regulatory roles in intestinal inflammatory injury, TLR4 (−8.2 kcal/mol) and HIF1A (−7.1 kcal/mol) were selected as candidate targets of AT-II. These two core target complexes were further selected for subsequent molecular dynamic simulations to explore their dynamic binding behaviors and structural stability under physiological conditions.

### 2.5. Molecular Dynamic Simulation Results

**Figure 4:**
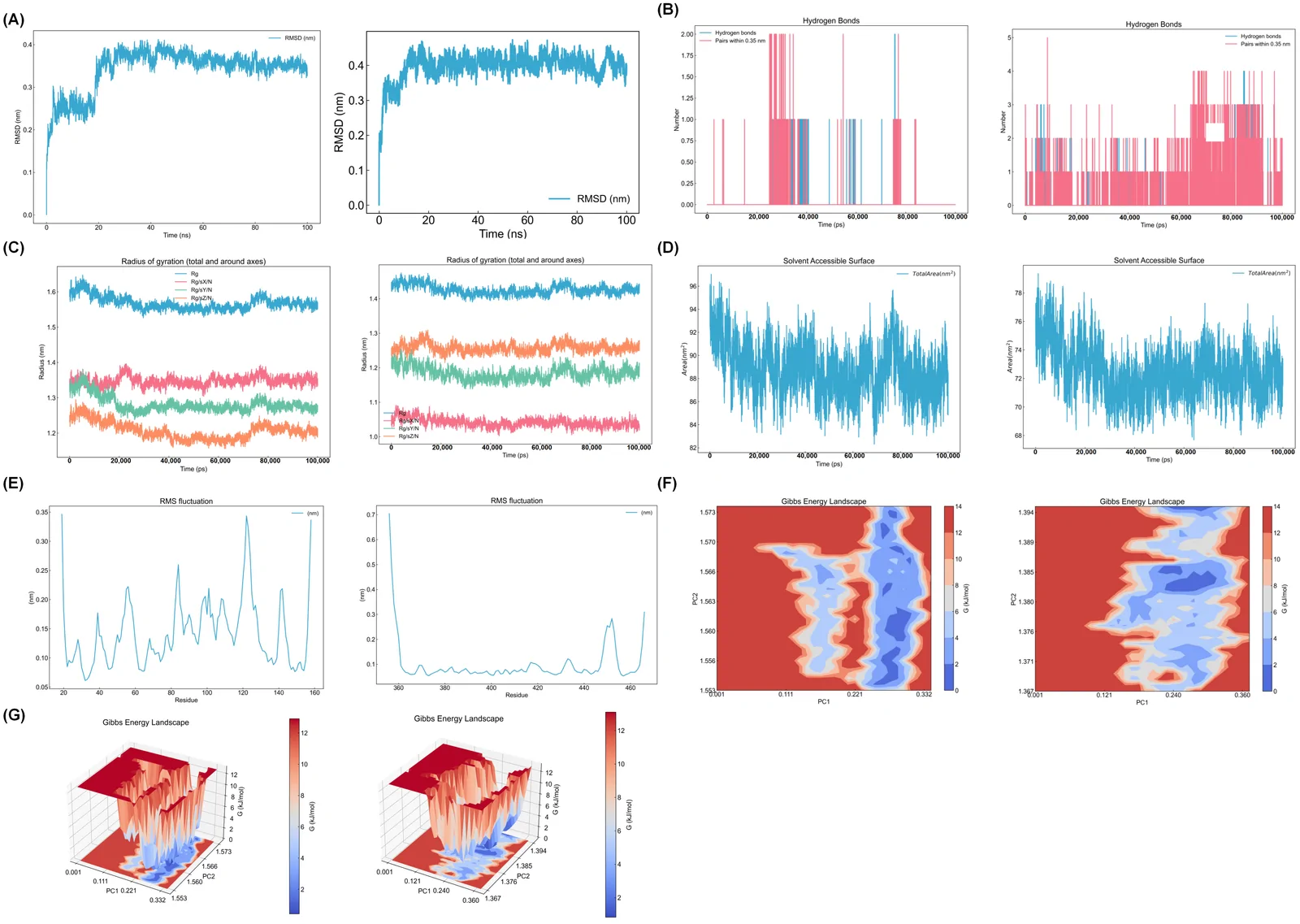
MD simulation between AT-II-TLII–TLR4 and AT-II-HIFII–HIF1A complexes. (**A**) RMSD results of AT-II in complex with TLR4 and HIF1A within 100 ns. (**B**) Hydrogen bonding number of AT-II complexed with TLR4 and HIF1A. (**C**) Rg results of AT-II in complex with TLR4 and HIF1A. (**D**) SASA results of AT-II in complex with TLR4 and HIF1A. (**E**) RMSF results of AT-II in complex with TLR4 and HIF1A. (**F**,**G**) Gibbs free-energy landscape (FEL) for the two complexes. Left, AT-II–TLR4 complex; right, AT-II–HIF1A complex.

The dynamic behaviors of the AT-II–TLR4 and AT-II–HIF1A complexes were simulated for 100 ns. Molecular dynamic simulations were applied to investigate the stability of the proteins and ligands during their interaction. This was performed by calculating several parameters, including RMSD, RMSF, hydrogen-bond occupancy, Rg, SASA, and FEL (Figure 4A–G).

For the AT-II–TLR4 complex, slight conformational rearrangement occurred within 18–20 ns, after which the RMSD stabilized at a plateau of 0.35–0.40 nm, indicating system equilibration. Intermolecular hydrogen bonds between AT-II and TLR4 were intermittent throughout the whole trajectory, suggesting that hydrophobic interactions and van der Waals forces may largely contribute to stable ligand–protein association. Rg and SASA suggested that the complex became slightly contracted at the early simulation stage and thereafter remained structurally compact. RMSF analysis characterized local residue flexibility; scattered fluctuation peaks existed along the protein sequence, whereas residues within the ligand-binding pocket did not exhibit excessive local flexibility, suggesting the ligand may be stably retained within the binding pocket. FEL analysis revealed two energy basins, with the system predominantly populated within the deeper basin, pointing toward a thermodynamically favored binding conformation for the AT-II–TLR4 complex.

In contrast, the RMSD values of the proteins fluctuated between 0.34 nm and 0.47 nm for AT-II–HIF1A, whereas persistent wide-range oscillation was observed across the entire trajectory. Notably, intermolecular hydrogen-bond contacts began to accumulate only after 65 ns, which suggests that the initial docking binding pose gradually deviated from the presumed binding pocket, and AT-II appeared to interact with surface regions of HIF1A. FEL analysis displayed a broad continuous low-energy channel instead of a well-confined dominant energy basin, suggesting that the complex sampled multiple interconvertible conformations and could not maintain a single stable binding conformation.

Collectively, MD simulation results suggest that the AT-II–TLR4 complex tends to show relatively superior conformational and binding stability compared with the AT-II–HIF1A complex. Guided by these computational findings, TLR4 was selected as the core target for subsequent assays.

### 2.6. DFT Calculation Results

**Figure 5:**
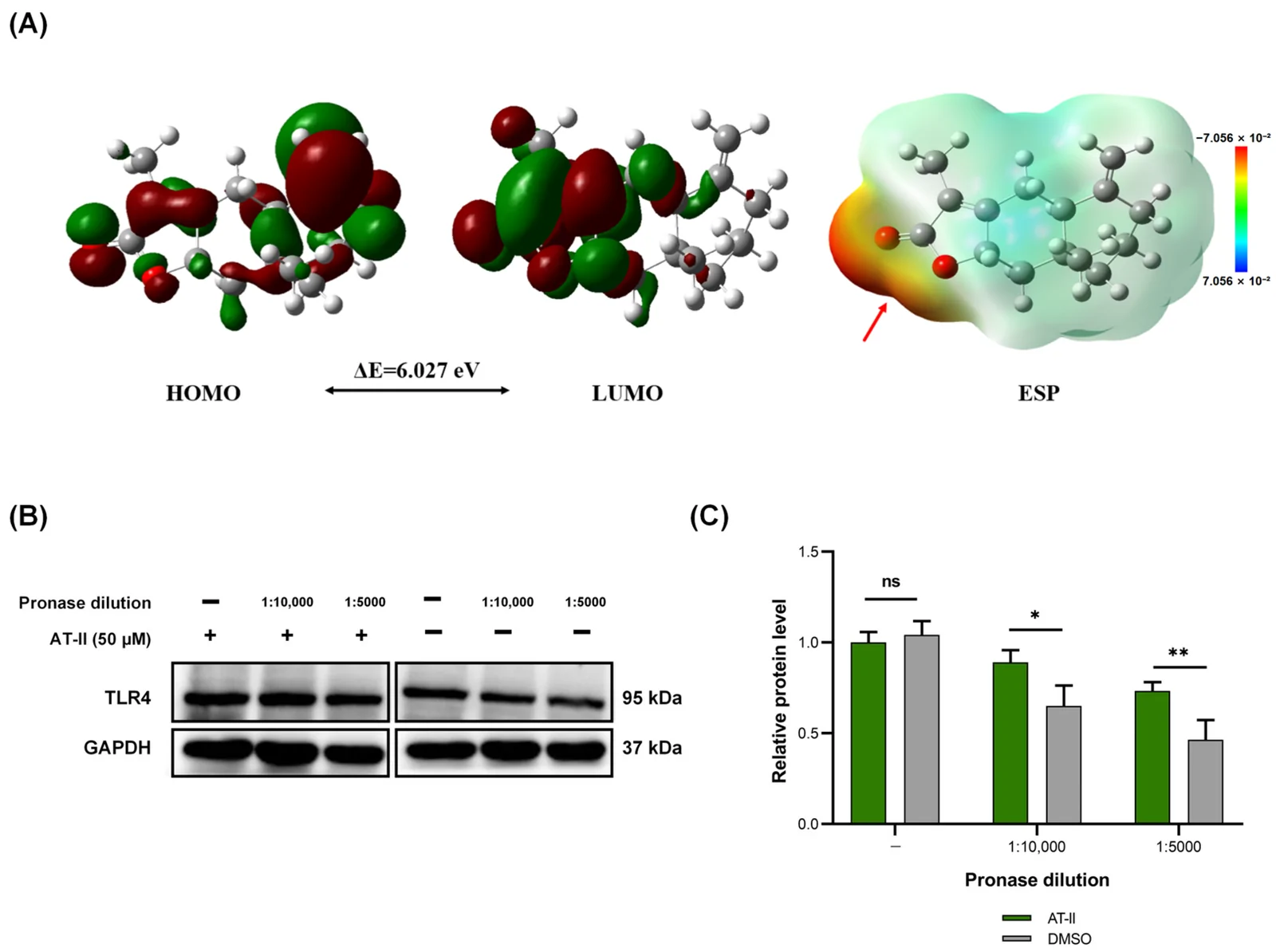
DFT calculation of AT-II and DARTS validation of its binding to TLR4. (**A**) Calculations of HOMO, LUMO and ESP for AT-II; (**B**) AT-II-enriched TLR4 protein was verified by DARTS (*n* = 3); (**C**) Quantitative analysis of TLR4 expression. ns, not significant; * *p* < 0.05, ** *p* < 0.01.

Density functional theory (DFT) calculations were performed to probe the electronic properties and potential intermolecular-interaction sites of AT-II (Figure 5A). A large HOMO–LUMO gap reflects favorable molecular stability, and this calculated 6.027 eV value suggests that AT-II may possess stable electronic-structural features while allowing feasible orbital interaction and electronic rearrangement. The HOMO and LUMO orbitals were predominantly localized on the lactone ring and its neighboring unsaturated carbon framework, suggesting this moiety as a potential key structural unit for electronic redistribution and orbital-mediated interactions [26,27]. ESP analysis revealed a heterogeneous charge distribution across the molecular surface. Strong negative electrostatic potential was observed near the carbonyl oxygen of the lactone ring, generating an electron-rich region capable of acting as a hydrogen-bond acceptor. This surface property may favor non-covalent interactions with amino acid residues within protein-binding pockets, providing a computational basis for plausible ligand–target intermolecular contacts.

### 2.7. DARTS Assay Supports a Potential Interaction Between AT-II and TLR4

To further characterize the binding between AT-II and TLR4 suggested by the preceding computational predictions, we employed a DARTS approach. This assay identifies direct targets for the ligand of interest without additional chemical modification and detects ligand-bound targets in cell lysates based on their increased resistance to proteolysis [20]. We incubated AT-II with IEC-6 cell lysates to test whether AT-II could engage TLR4.

No obvious difference in TLR4 protein degradation was observed between control and AT-II-treated samples in the absence of pronase digestion. Upon pronase exposure, TLR4 from AT-II-treated lysates exhibited markedly enhanced resistance to proteolytic degradation. Significant protective effects were detected at pronase dilution ratios of 1:10,000 (*p* < 0.05) and 1:5000 (*p* < 0.01) (Figure 5B,C). These findings support a potential interaction between AT-II and TLR4 at the cellular protein level. These observations support our hypothesis and facilitate subsequent mechanistic investigations.

### 2.8. Effects of AT-II on 5-FU-Induced TLR4/MYD88/NF-κB Signaling in IEC-6 Cells

TLR4 plays a key role in mediating intestinal inflammatory responses, and MYD88 acts as an intermediate linker for the downstream inflammatory cascade, which is critical for 5-FU-induced intestinal epithelial injury [6,7]. Building upon these findings, we further carried out cellular assays to evaluate the protective effects of AT-II against 5-FU-induced intestinal epithelial injury in IEC-6 cells and its regulatory effects on the TLR4/MYD88/NF-κB signaling pathway [1,9].

**Figure 6:**
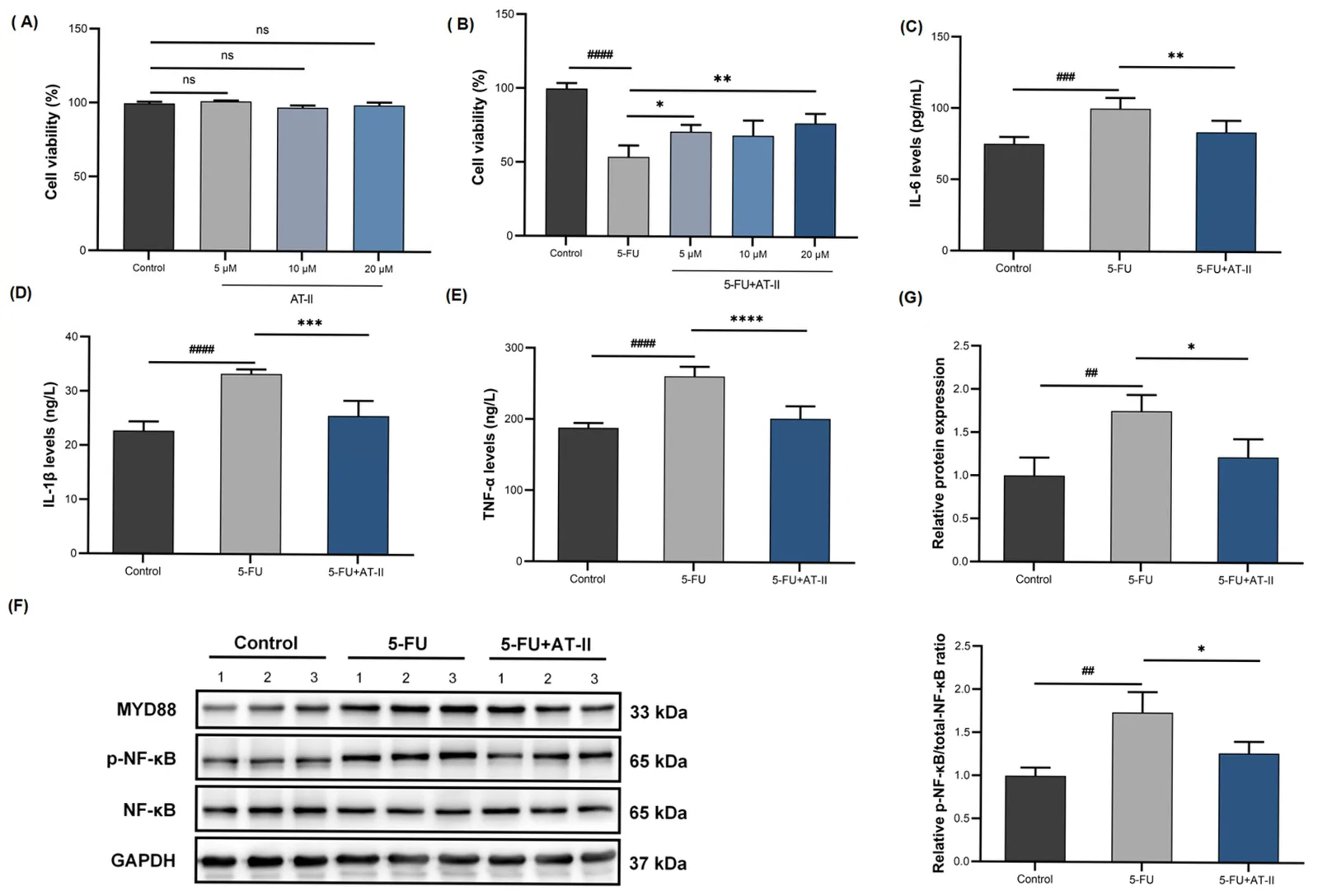
AT-II alleviates 5-FU-induced inflammatory injury and regulates MYD88/NF-κB signaling in IEC-6 cells. (**A**) Cell viability of IEC-6 cells treated with 5, 10, 20 μM AT-II assessed by the CCK-8 assay (*n* = 3). (**B**) Cell viability assessed by the CCK-8 assay (*n* = 4). (**C**–**E**) Levels of pro-inflammatory cytokines IL-6, IL-1β, and TNF-α determined by ELISA (*n* = 5). (**F**) Representative Western blot bands for MYD88, p-NF-κB, total NF-κB, and GAPDH. (**G**) Quantitative analysis of MYD88 expression and the p-NF-κB/total NF-κB ratio (*n* = 3). ^##^ *p* < 0.01, ^###^ *p* < 0.001, ^####^ *p* < 0.0001 compared to the control group; * *p* < 0.05, ** *p* < 0.01, *** *p* < 0.001, **** *p* < 0.0001 compared to the 5-FU-treated group; ns, not significant.

The CCK-8 assay was applied to verify the protective effect of AT-II and identify its working concentration. As shown in Figure 6A, treatment with 5, 10, or 20 μM AT-II did not significantly alter cell viability compared with the control group, indicating that AT-II at the working concentration used in this study exhibits no obvious cytotoxicity toward IEC-6 cells, while 75 μM 5-FU markedly reduced the viability of IEC-6 cells (*p* < 0.0001). Co-treatment with 5 μM and 20 μM AT-II significantly restored cell viability (*p* < 0.05 and *p* < 0.01, respectively), whereas 10 μM AT-II yielded no significant improvement in cell viability. Non-monotonic dose–response effects are common among sesquiterpenes. Based on these observations, 20 μM AT-II was chosen for subsequent assays (Figure 6B).

To assess the anti-inflammatory potential of AT-II, we measured the secretion of pro-inflammatory cytokines via ELISA. 5-FU challenge markedly increased IL-1β, IL-6 and TNF-α levels (*p* < 0.001, *p* < 0. 001 and *p* < 0.0001, respectively) in IEC-6 cells. Following treatment with 20 μM AT-II, these elevated cytokine levels were substantially suppressed (Figure 6C–E). These observations indicated that AT-II could effectively reduce excessive inflammatory activation triggered by 5-FU in IEC-6 cells.

We further performed Western blot to detect changes in the MYD88/NF-κB pathway. 5-FU treatment significantly upregulated MYD88 expression and increased NF-κB p65 phosphorylation (*p* < 0.01, *p* < 0.01). Upon AT-II treatment, these increased MYD88 and p-NF-κB p65 levels were significantly reversed (*p* < 0.05, *p* < 0.05). Notably, total NF-κB levels showed no marked differences among groups. These findings suggested that AT-II regulates the TLR4 downstream MYD88/NF-κB pathway mainly by suppressing NF-κB p65 phosphorylation, rather than altering total NF-κB levels.

**Figure 7:**
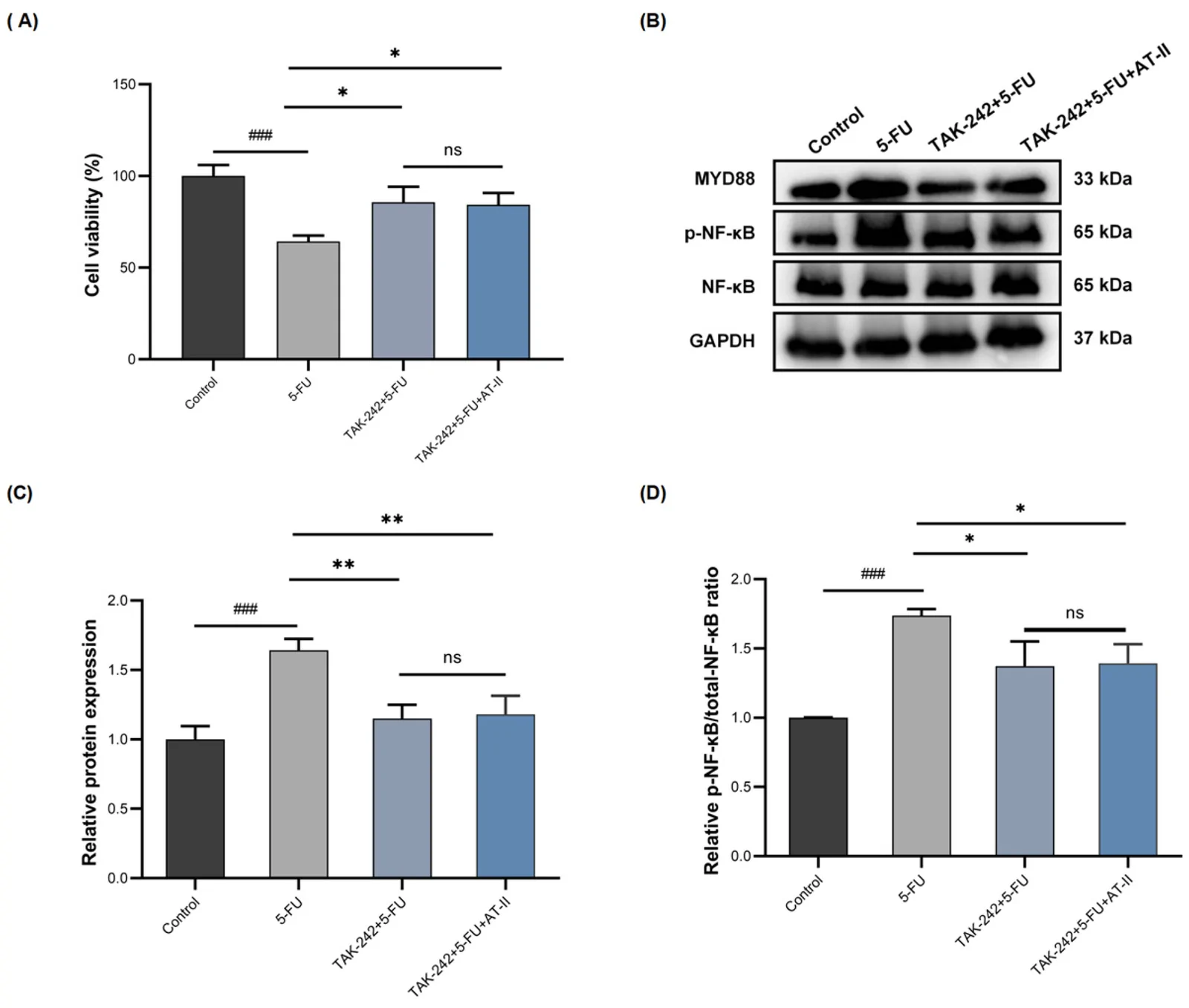
TAK-242 inhibition of TLR4 attenuates the protective effect of AT-II against 5-FU-induced intestinal injury in IEC-6 cells. (**A**) Cell viability assessed by the CCK-8 assay (*n* = 3). (**B**) Representative Western blot bands for MYD88, p-NF-κB, total NF-κB, and GAPDH. (**C**,**D**) Quantitative analysis of MYD88 expression and the p-NF-κB/total NF-κB ratio (*n* = 3). ^###^ *p* < 0.001 compared to the control group; * *p* < 0.05, ** *p* < 0.01 compared to the 5-FU-treated group; ns, not significant.

To further examine whether the protective effect of AT-II depends on TLR4, additional experiments were performed using the TLR4 inhibitor TAK-242. As shown in Figure 7A, 5-FU exposure markedly reduced IEC-6 cell viability. Pretreatment with TAK-242 restored cell viability following 5-FU injury; however, AT-II co-treatment produced no further improvement in cell survival.

Similarly, Western blot results showed that 5-FU exposure increased MYD88 and p-NF-κB p65 levels. Inhibition of TLR4 by TAK-242 reversed these changes. No additional reduction in MYD88 or p-NF-κB p65 levels was observed when AT-II was added alongside TAK-242 and 5-FU. Taken together, these cellular data suggested that AT-II ameliorates 5-FU-induced intestinal epithelial injury potentially via targeting TLR4 and suppressing the MYD88/NF-κB signaling pathway.

## 3. Discussion

5-Fluorouracil (5-FU) is a first-line fluoropyrimidine antimetabolite widely used for the clinical management of gastrointestinal malignancies. However, its clinical application is greatly limited by dose-dependent toxicity, among which intestinal toxicity represents a major dose-limiting adverse reaction [1,2,3]. In vivo, 5-FU preferentially damages rapidly proliferating intestinal epithelial cells, triggering excessive inflammatory activation and intestinal barrier dysfunction [6]. At present, clinical management of 5-FU-induced intestinal toxicity relies largely on symptomatic treatment and empirical dose adjustment, with few specific and effective protective interventions available. Natural bioactive compounds derived from traditional Chinese medicine exhibit favorable safety profiles and multitargeted regulatory capacities, making them ideal adjuvant candidates for chemotherapy protection [13,14]. Accordingly, screening and validating effective natural agents is critical for the mitigation of 5-FU-induced intestinal toxicity.

Atractylodis Rhizoma, a traditional Chinese medicinal herb, is applied to treat gastrointestinal ailments. Preparations derived from this herb reportedly alleviate chemotherapy-induced gastrointestinal injury. Phytochemical studies have identified sesquiterpenes as its primary bioactive components, conferring intestinal protection and anti-inflammatory properties [11,17]. Atractylenolide II (AT-II), a characteristic sesquiterpene lactone from Atractylodis Rhizoma, exerts multiple biological activities, including anti-tumor, anti-inflammatory, antioxidant and cytoprotective effects [15,16,17,28]. Available reports have shown that AT-II suppresses tumor cell proliferation and promotes apoptosis through several signaling pathways such as PADI3-ERK, JAK2/STAT3 and Akt/ERK pathways [16,29,30]. In addition to its anti-tumor effects, AT-II protects normal cells from oxidative injury induced by radiation via modulation of the MAPKp38/Nrf2 pathway, supporting its cytoprotective effects in non-neoplastic cells [15]. Nevertheless, whether AT-II mitigates 5-FU-triggered intestinal toxicity and the underlying molecular mechanisms remain undefined. In this work, UHPLC-QE-MS was applied to characterize the chemical composition of Atractylodis Rhizoma, and AT-II was selected as the key bioactive constituent for mechanistic investigation [11]. In silico ADMET assessment suggests that AT-II satisfies druglikeness criteria and may exhibit favorable oral absorption potential. These predicted properties are consistent with reported in vivo pharmacokinetic behavior following oral administration of Atractylodis Rhizoma [25]. Such predicted druglikeness supports its potential as an intestinal protective agent, with the caveat of a predicted CYP2C19 interaction liability.

Network pharmacology offers a systematic approach to dissect multi-target and multi-pathway regulation by natural compounds [18,19]. In this work, we identified 32 overlapping targets linked to AT-II and 5-FU-induced intestinal toxicity. Hub genes were enriched in pathways related to intestinal inflammation and stress responses, known to mediate 5-FU-induced intestinal toxicity. Consistent with prior studies on 5-FU intestinal injury, overactive inflammatory signaling drove intestinal epithelial apoptosis and mucosal barrier disruption [1,4,6]. These data suggest that AT-II may mitigate 5-FU-induced intestinal toxicity via modulation of inflammatory and stress-related pathways and guided our subsequent target validation and functional assays.

Molecular docking was used to assess the static binding affinity between AT-II and hub target proteins. All core targets exhibited favorable binding affinity for AT-II. Based on binding energy, network topology, pathway enrichment results and disease association, TLR4 and HIF1A were selected as candidate targets. Since static docking does not capture dynamic conformational alterations of ligand–protein complexes under physiological conditions, a 100 ns MD simulation was carried out to compare binding stability of the two candidate complexes. The simulations suggested that the AT-II–TLR4 complex retained a stable backbone and compact conformation throughout the simulation, whereas the AT-II–HIF1A complex displayed marked conformational drift and unstable binding. These MD simulations support the possibility that AT-II forms a more stable complex with TLR4.

DFT calculations provide further theoretical clues for the favorable binding of AT-II with TLR4. The lactone ring of AT-II exhibits an electron-rich region and acts as a key hydrogen-bond acceptor, offering potential non-covalent interaction sites for contacts with amino acid residues in TLR4. This feature agrees well with previously reported structure–activity relationships (SAR) for sesquiterpene lactones [26,27]. Prior work indicates that the lactone skeleton serves as the core functional motif of sesquiterpene lactones, and this lactone moiety is important for suppressing the TLR4-mediated signaling pathway [26]. Similarly, other lactone-containing natural anti-inflammatory compounds exert TLR4-directed anti-inflammatory activity via their lactone ring. These findings further support our computational results and offer molecular hints for the proposed interaction between AT-II and TLR4 to produce anti-inflammatory effects [27].

Computational simulations only provide predictive evidence and cannot conclusively verify molecular interactions. Accordingly, this study adopted the label-free DARTS assay to explore the physical interaction between AT-II and TLR4 [20]. Unlike conventional labeling-dependent verification approaches that may interfere with intrinsic biological properties, DARTS enables the direct detection of small molecule–protein binding in native cell lysates without compound modification, making it well suited for target validation of natural bioactive compounds [21,22]. The core principle of DARTS is that stable ligand–protein binding protects target proteins from enzymatic hydrolysis. Consistent with this mechanism, our results showed that AT-II treatment increased TLR4 protein stability in IEC-6 cell lysates, supporting a potential physical interaction between AT-II and TLR4.

In vitro cellular assays further characterized AT-II’s protective action and downstream signaling regulation. 5-FU treatment reduced viability and triggered inflammatory injury in IEC-6 cells, accompanied by elevated secretion of pro-inflammatory cytokines IL-1β, IL-6 and TNF-α. This phenotype reproduces key pathological features of 5-FU-induced intestinal epithelial injury [1,9]. AT-II treatment restored cell viability and suppressed the increased release of these pro-inflammatory cytokines. Mechanistically, AT-II inhibited activation of the TLR4/MYD88/NF-κB signaling pathway [31]. Consistent with our Western blot data, AT-II reduced NF-κB p65 phosphorylation without changing total NF-κB p65 levels, suggesting that AT-II modulates NF-κB-driven inflammation via phosphorylation. This mode of regulation aligns with known responses to 5-FU: 5-FU activates upstream TLR4/MYD88 signaling to drive p65 phosphorylation and amplify inflammatory signaling, while total NF-κB protein expression remains unaltered [6,7].

Numerous studies have demonstrated that suppression of the TLR4/MYD88/NF-κB signaling pathway represents an effective strategy for ameliorating 5-FU-induced intestinal toxicity [5,6,7]. Many compounds derived from traditional Chinese medicine reduce intestinal inflammatory injury through this axis [32]. It has also been reported that AT-II can modulate TLR4-related inflammatory responses. However, most prior work relies solely on cellular phenotypic and protein expression data, without target-binding validation or structural mechanistic interpretation. In contrast to earlier purely phenotypic studies, our work integrated multiple computational pharmacology approaches with the DARTS assay and TLR4 inhibitor treatment [33,34], which showed that the protective effect of AT-II was largely lost when TLR4 was blocked, supporting the hypothesis that AT-II may interact with TLR4 and suppress the downstream MYD88/NF-κB signaling pathway.

This multi-layered experimental and computational strategy notwithstanding, the present study has two main limitations. First, all functional assays were performed using the IEC-6 cell line. In vivo animal models would be needed to validate AT-II’s protective effects against 5-FU-induced intestinal toxicity. Second, our investigation focused primarily on the TLR4/MYD88/NF-κB signaling pathway. Although MD simulations indicated unstable binding between AT-II and HIF1A, we cannot rule out potential regulatory effects of AT-II on HIF-1 signaling or other hub targets. These possibilities warrant further investigation in future work.

## 4. Materials and Methods

### 4.1. Chemicals and Reagents

Atractylenolide II (AT-II, purity ≥ 98%) was purchased from Chengdu Push Bio-Technology Co., Ltd. (Chengdu, China). 5-Fluorouracil (5-FU) was obtained from MedChemExpress (Shanghai, China). LC-MS-grade methanol, acetonitrile, and formic acid were supplied by Thermo Fisher Scientific (Waltham, MA, USA). Fetal bovine serum (FBS), Dulbecco’s modified Eagle’s medium (DMEM), insulin, and penicillin–streptomycin were purchased from Meilun Biotechnology (Dalian, China). Primary antibodies against MYD88 (1:1000), TLR4 (1:300), and total NF-κB p65 (1:1000) were obtained from Selleck Chemicals (Houston, TX, USA). The primary antibody against p-NF-κB p65 (1:1000) and GAPDH (1:8000) were purchased from Proteintech Group (Wuhan, China). HRP-conjugated secondary antibodies were also acquired from the same vendor. All other analytical-grade reagents were utilized directly without additional purification.

### 4.2. Optimization of UPLC-MS/MS Conditions

Atractylodis Rhizoma was collected from Luotian County (Huanggang, Hubei Province, China) and identified as the dried rhizome of Atractylodes lancea (Thunb.) DC. by the Department of TCM Identification, Liaoning University of Traditional Chinese Medicine. The chemical constituents of Atractylodis Rhizoma were analyzed using a Vanquish Duo UHPLC system coupled with a Q Exactive high-resolution mass spectrometer (Thermo Fisher Scientific, Waltham, MA, USA). Briefly, 0.5 g of dried Atractylodis Rhizoma powder was ultrasonically extracted with 25 mL of 90% methanol for 45 min (250 W, 50 kHz). The mixture was then centrifuged at 12,000 rpm for 10 min. The resulting supernatant was filtered using a 0.22 μm syringe filter prior to instrumental analysis.

Chromatographic separation was carried out on a Waters HSS T3 C18 column (2.1 mm × 150 mm, 1.7 μm). The mobile phase consisted of ultrapure water with 0.1% methanoic acid (solution A) and acetonitrile (solution B). The gradient elution was set as follows: 0–2.0 min, 10% B; 2.0–22.0 min, 10–56% B; 22.0–52.0 min, 56–80% B; 52.0–55.0 min, 80–95% B; 55.0–58.0 min, 95% B. The flow rate was 0.20 mL/min, the column temperature was maintained at 45 °C, and the injection volume was 2 μL.

Mass-spectrometric detection was performed in both positive- and negative-ion heated electrospray ionization (HESI) modes. The spray voltage was set to 3.5 kV for positive-ion mode and 4.0 kV for negative-ion mode. The capillary temperature was 320 °C, sheath-gas flow was 35 arb, and auxiliary-gas flow was 10 arb. Full-scan and data-dependent MS^2^ (dd-MS^2^) acquisition modes were applied, with mass resolutions of 70,000 for full-scan MS and 17,500 for MS^2^. The mass scan range was set at m/z 100–1500, and normalized collision energies (NCE) were adjusted to 20%, 30%, and 40%. Xcalibur 4.1 software was used to acquire raw data. Compound annotation was achieved by comparing retention times, accurate molecular masses, and MS^2^ fragment patterns against published data from relevant references.

### 4.3. ADMET and Druglikeness Analysis

In silico profiling of absorption, distribution, metabolism, excretion, and toxicity (ADMET) parameters together with druglike characteristics of AT-II were carried out using the ADMETlab 3.0 web server (https://admetlab.scbdd.com/). The computed descriptors included fundamental physicochemical properties, gastrointestinal absorption potential, P-glycoprotein substrate liability, cytochrome P450-related metabolic patterns, plasma pharmacokinetic parameters, and hERG-linked cardiac safety profiles. Druglikeness was assessed based on Lipinski’s rule of five and other standard medicinal chemistry criteria.

### 4.4. Network Pharmacology Analysis

The SMILES string of AT-II was obtained from the PubChem database (https://pubchem.ncbi.nlm.nih.gov/). Candidate molecular targets of AT-II were predicted by means of SwissTargetPrediction (https://swisstargetprediction.ch/, accessed on 3 August 2026), with a probability cutoff set to ≥0.1. Disease targets associated with “5-FU-induced intestinal toxicity” were compiled from the GeneCards (https://www.genecards.org/) and OMIM (http://www.omim.org/) databases. The Venn diagram tool 2.1.0 (https://bioinfogp.cnb.csic.es/tools/venny/, accessed on 3 August 2026) was utilised to identify intersecting targets shared by AT-II and 5-FU-induced intestinal toxicity.

These intersecting targets were uploaded to the STRING database (http://string-db.org/) to build a protein–protein interaction (PPI) network. *Homo sapiens* was selected as the organism filter, while all other parameters remained at default values. PPI datasets were imported into Cytoscape 3.10.2 for visualization, and the Centiscape 2.2 plugin was applied for topological calculation. Core targets were prioritized on the basis of degree, betweenness centrality, and closeness centrality.

Enrichment assessments, covering Gene Ontology (GO) and Kyoto Encyclopedia of Genes and Genomes (KEGG) pathway analysis, were carried out for the intersecting targets using the DAVID database (https://davidbioinformatics.nih.gov/).The GO enrichment were classified into three subgroups: biological process (BP), cellular component (CC), and molecular function (MF). Terms and pathways with *p* < 0.05 were taken as statistically significant. The most highly enriched entries were visualized with the aid of an online bioinformatics platform (http://www.bioinformatics.com.cn/).

### 4.5. Molecular Docking

The three-dimensional structure of AT-II was retrieved from PubChem and geometrically optimized using Chem3D. The crystal structures of the ten hub targets (TLR4, PDB: 4G8A; HIF1A, PDB: 4H6J; ESR1, PDB: 1XPC; PPARG, PDB: 3ADX; PTGS2, PDB: 5F1A; IL1B, PDB: 2NVH; STAT3, PDB: 9RL6; NFKB1, PDB: 8TQD; BCL2L1, PDB: 4QVF; HSP90AA1, PDB: 3B24) were downloaded from the RCSB Protein Data Bank.

PDB 4G8A is the TLR4–MD-2 complex bearing the D299G/T399I polymorphism, which features a well-resolved MD-2 ligand-binding pocket. PDB 4H6J corresponds to the HIF-1α–ARNT PAS-B heterodimer. Since high-resolution crystal structures of full-length HIF1A are unavailable, the PAS-B domain was selected as it is important for this domain heterodimerization and allosteric regulation. For these two structures, only chains A and C of 4G8A and chains A and B of 4H6J were retained, while redundant chains were discarded.

In PyMOL 2.3.0, crystallized water molecules, exogenous ligands and redundant atoms were removed. Ligand and receptor structures were prepared, partial charges were assigned, and files were exported as pdbqt files in AutoDockTools 1.5.7. Grid boxes covering the full receptor structure were defined. Rigid molecular docking was carried out using AutoDock Vina 1.1.2, and optimal binding poses were visualized in PyMOL.

### 4.6. Molecular Dynamic Simulation

To characterize the conformational features and binding stability of the docked AT-II–TLR4 and AT-II–HIF1A complexes, GROMACS 2020 was utilised for molecular dynamics simulations. Protein topology files were built adopting the Amber99SB-ILDN force field through the pdb2gmx module, whereas ligand topologies were generated by means of ACPYPE 2023. The TIP3P water model was adopted for system construction, and counter-ions were supplemented to neutralize the net charge of the aqueous system. Energy minimization was implemented to refine atomic coordinates. Two sequential equilibration runs of 100 ps duration, NVT followed by NPT ensemble, were executed to stabilize system temperature and pressure. Subsequently, the production MD run was extended to 100 ns, with a 2 fs integration timestep. After simulation, trajectory files were processed to correct the periodic boundary condition (PBC). RMSD, RMSF, radius of gyration (Rg), and free-energy landscape (FEL) analyses were then carried out to assess the dynamic behavior of these protein–ligand complexes.

### 4.7. DFT Calculations

DFT calculations were implemented using Gaussian 09W. The molecular geometry of AT-II underwent full geometry optimization at the B3LYP/6-31G(d) theoretical level. Frontier molecular orbital (HOMO and LUMO) energies together with their associated energy gap were determined. Electrostatic potential (ESP) maps were rendered via GaussView to examine the chemical reactivity and charge-distribution properties of AT-II.

### 4.8. Drug Affinity-Responsive Target Stabilization Assay (DARTS)

The DARTS assay was conducted according to the literature with minor modifications in IEC-6 cells [21]. In brief, approximately 1 × 10^7^ untreated IEC-6 cells were lysed with a mammalian protein extraction reagent on ice for 30 min. Total protein was harvested by centrifugation and quantified using the BCA assay. Then, protein lysates were incubated with 50 μM AT-II or an equal volume of DMSO vehicle for 2 h at room temperature to allow sufficient ligand–protein target binding. Samples were gently mixed during incubation.

Subsequently, pronase digestion at dilution ratios of 1:10,000 and 1:5000 was carried out at 37 °C for 30 min. Then, loading buffer was added to the lysates, and the samples were boiled for 10 min. The resulting samples were separated by sodium dodecyl sulfate–polyacrylamide gel electrophoresis (SDS-PAGE). All DARTS experiments were performed in three independent biological replicates.

### 4.9. Cell Culture and Treatment

The IEC-6 cell line was obtained from Shanghai Gaining Biological Technology Co., Ltd. (Shanghai, China). IEC-6 cells were cultured in DMEM medium containing 10% FBS, 0.01 mg/mL insulin, and 1% penicillin–streptomycin. The cells were incubated at 37 °C under 5% CO_2_. To assess the effect of AT-II on cell viability, additional groups treated with 5, 10 and 20 μM AT-II alone for 24 h were included. Cells were divided into five groups: control group, 5-FU-treated group (75 μM 5-FU for 24 h), and co-treatment groups with 75 μM 5-FU plus 5 μM, 10 μM or 20 μM AT-II for 24 h. For TLR4 inhibition assays, IEC-6 cells were pretreated with 25 μM TAK-242 (Selleck Chemicals, Houston, TX, USA) for 2 h before exposure to 75 μM 5-FU, with or without 20 μM AT-II. Four experimental groups were set: control, 5-FU, TAK-242 + 5-FU, and TAK-242 + 5-FU + AT-II. All treatments were maintained for 24 h.

### 4.10. Cell Viability Assay

The viability of IEC-6 cells was evaluated via the CCK-8 assay. Briefly, IEC-6 cells were seeded into 96-well plates and subjected to the corresponding treatments for 24 h. Afterwards, 10 µL of CCK-8 reagent was supplemented, followed by incubation at 37◦C for 2 h. Optical density values were recorded at 450 nm with a microplate reader.

### 4.11. ELISA Assay for Determination of Cytokine Levels

After cell treatment, the cell culture supernatant was collected. The supernatant was briefly centrifuged, aliquoted, and stored at −80 °C before detection. The concentrations of pro-inflammatory cytokines (IL-1β, IL-6, TNF-α) were detected using commercial ELISA kits (TNF-α: MM-0180R1; IL-6: MM-0190R1; IL-1β: MM-0047R1, Jiangsu Meimian Industrial Co., Ltd., Yancheng, China) according to the manufacturer’s instructions.

### 4.12. Western Blot Analysis

Cells were lysed by shaking in RIPA lysis buffer supplemented with protease inhibitors. Total protein content was quantified via the BCA method. Cell extract (20 µg) was applied to SDS-PAGE, transferred to PVDF membranes, and subsequently blocked with 5% skim milk in TBST for 2 h. The membranes were incubated at 4 °C with primary antibodies (TLR4, MYD88, total NF-κB, p-NF-κB, and GAPDH) overnight. On the next day, the membranes were rinsed three times (10 min per wash) in TBST before incubation with corresponding secondary antibodies for 1 h. Protein bands were detected using an ECL reagent kit, and band intensities were quantified with ImageJ 1.52a.

### 4.13. Statistical Analysis

All data are expressed as means ± standard deviation (SD). Statistical evaluations were carried out in GraphPad Prism 8.0. One-way analysis of variance (ANOVA) was adopted, accompanied by Tukey’s multiple comparison test for intergroup comparisons. *p* < 0.05 was considered statistically significant.

## 5. Conclusions

In summary, this in vitro study indicates that AT-II exerts protective effects against 5-FU-induced intestinal toxicity potentially via targeting TLR4 and the downstream MYD88/NF-κB signaling pathway. Network pharmacology and molecular docking predicted TLR4 as a potential target, which was further supported by molecular dynamic simulation and DFT calculation, and consistent with the results of the DARTS assay. TLR4 inhibitor experiments further suggested this protective action is linked to TLR4 signaling. Cellular experiments suggested that AT-II alleviates 5-FU-triggered inflammatory injury. These preliminary in vitro findings suggest that AT-II might represent a promising candidate for further investigation into mitigating chemotherapy-associated intestinal toxicity.

## Figures and Tables

**Table 1:** ADMET and druglikeness prediction results of AT-II.

**Physicochemical properties**	MW	nHA	nHD	TPSA	Log P
	232.15	2.0	0.0	26.3	2.938
**Druglikeness rules**	Lipinski rule	Pfizer rule	GSK rule	Golden triangle	
	accepted	accepted	accepted	accepted	
**Absorption**	Caco-2 permeability	Pgp substrate	HIA	F_20%_	F_30%_
	−4.476	0.048 (---)	0.001 (---)	0.094 (---)	0.259 (--)
**Distribution**	PPB	VDss			
	96.027%	0.065 L/kg			
**Metabolism**	CYP1A2 inhibitor	CYP2C9 inhibitor	CYP2C19 inhibitor	CYP2D6 inhibitor	CYP3A4 inhibitor
	0.003 (---)	0.002 (---)	0.901 (+++)	0.0 (---)	0.141 (--)
	CYP1A2 substrate	CYP2C9 substrate	CYP2C19 substrate	CYP2D6 substrate	CYP3A4 substrate
	0.685 (+)	0.987 (+++)	0.999 (+++)	0.801 (++)	0.527 (+)
**Excretion**	Plasma clearance	T_1/2_			
	9.987 mL/min/kg	1.318 h			
**Toxicity**	hERG blocker				
	0.026 (---)				

Prediction probability ranges: 0–10% (---), 10–30% (--), 50–70% (+), 70–90 (++), and 90–100% (+++). MW: molecular weight; TPSA: topological polar surface area; nHA: number of hydrogen bond acceptors; nHD: number of hydrogen bond donors; HIA: probability of poor human intestinal absorption; Fa_20%_/Fa_30%_: 20%/30% bioavailability; PPB: plasma protein binding; VDss: volume distribution.

**Table 2:** Binding affinity (kcal/mol) between AT-II and the key targets.

Target	Target (PDB ID)	Affinity (kcal/mol)
TLR4	4G8A	−8.2
HIF1A	4H6J	−7.1
ESR1	1XPC	−9.1
PPARG	3ADX	−7.3
PTGS2	5F1A	−7
IL1B	2NVH	−6.9
STAT3	9RL6	−6.4
NFKB1	8TQD	−6.3
BCL2L1	4QVF	−6.3
HSP90AA1	3B24	−6.1

## Data Availability

The original contributions presented in this study are included in the article/Supplementary Materials. Further inquiries can be directed to the corresponding authors.

## Supplementary Materials

The following supporting information can be downloaded at https://www.mdpi.com/article/10.3390/molecules31213968/s1. Figure S1: Identified chemical constituents in Atractylodis Rhizoma; Table S1: Identified chemical constituents in Atractylodis Rhizoma.

## Abbreviations

The following abbreviations are used in this manuscript.

5-FU	5-fluorouracil
AT-II	atractylenolide II
PPI	protein–protein interaction
GO	Gene Ontology
KEGG	Kyoto Encyclopedia of Genes and Genomes
BP	biological process
MF	molecular function
CC	cellular component
DFT	density functional theory
HOMO	highest occupied molecular orbital
LUMO	lowest unoccupied molecular orbital
ESP	electrostatic potential
DARTS	drug affinity-responsive target stability
TLR4	Toll-like receptor 4
MYD88	myeloid differentiation factor 88
NF-κB	nuclear factor kappa B
IL-1β	interleukin 1β
IL-6	interleukin 6
TNF-α	tumor necrosis factor α
