# Supplementary material for: Atractylenolide II Alleviates 5-Fluorouracil-Induced Intestinal Toxicity Through Potentially Targeting the TLR4-Mediated MYD88/NF-κB Signaling Pathway: An Integrated In Silico and In Vitro Study
Source: Molecules. 2026 Nov 3;31(21):3968. doi: 10.3390/molecules31213968 (PMC13765098; doi:10.3390/molecules31213968)
Supplement: Supplementary file 1 [file molecules-31-03968-s001.zip › molecules-4588081-supplementary.pdf]

Supplementary Materials:

Table S1. Identified chemical constituents in Atractylodis Rhizoma.

NO.	tR/min	Obsed /(m/z)	Calcd /(m/z)	Adducts	Error /(ppm)	Fragments	Formula	Identity	Classification
1	1.26	133.0141	133.0137	$[M-H]^-$	3.01	115.0035, 89.0243, 71.0138	$C_4H_6O_5$	Malic acid	Organic acids
2	1.30	191.0560	191.0556	$[M-H]^-$	2.28	173.0444, 127.0412, 111.0460	$C_7H_{12}O_6$	Quinic acid	Organic acids
3	1.42	182.0811	182.0817	$[M+H]^+$	-3.54	165.0701	$C_9H_{11}NO_3$	Tyrosine	Amino acids
4	1.77	353.0875	353.0873	$[M-H]^-$	0.68	191.0559, 179.0348, 161.0239, 135.0453	$C_{16}H_{18}O_9$	Chlorogenic acid	Organic acids
5	1.88	353.0877	353.0873	$[M-H]^-$	1.25	191.0559, 179.0348, 173.0453, 161.0239, 135.0453	$C_{16}H_{18}O_9$	Cryptochlorogenic acid	Organic acids
6	1.96	447.2236	447.2230	$[M-H]^-$	1.29	285.1705, 267.1600, 249.1491, 179.0553, 161.0454	$C_{21}H_{36}O_{10}$	Atractyloside A	Sesquiterpenes
7	2.20	167.0349	167.0344	$[M-H]^-$	2.78	152.0114, 123.0451, 108.0216	$C_8H_8O_4$	Vanillic acid	Organic acids
8	2.30	367.1033	367.1029	$[M-H]^-$	1.06	193.0504, 191.0559, 173.0453, 149.0605, 134.0372	$C_{17}H_{20}O_9$	3-O-Feruloylquinic Acid	Organic acids
9	4.87	193.0506	193.0501	$[M-H]^-$	2.67	178.0271, 149.0606, 134.0372	$C_{10}H_{10}O_4$	Ferulic acid	Organic acids
10	6.44	515.1191	515.1190	$[M-H]^-$	0.28	353.0876, 191.0560, 179.0348, 161.0239, 135.0405	$C_{25}H_{24}O_{12}$	Isochlorogenic acid A	Organic acids
11	6.93	193.0495	193.0501	$[M+H]^+$	-3.03	178.0270, 149.0607, 134.0372	$C_{10}H_8O_4$	Scopoletin	Coumarins
12	9.87	575.2711	575.2704	$[M-H]^-$	1.27	529.2650, 113.0243	$C_{27}H_{44}O_{13}$	Atractyloside I	Sesquiterpenes
13	10.86	515.1195	515.1190	$[M-H]^-$	1.06	353.0866, 191.0557, 179.0347, 173.0454, 161.0245, 135.0405	$C_{25}H_{24}O_{12}$	Isochlorogenic acid C	Organic acids
14	12.29	221.1899	221.1905	$[M+H]^+$	-2.89	203.1795, 161.1326	$C_{15}H_{24}O$	Spathulenol	Sesquiterpenes
15	12.84	415.2340	415.2332	$[M-H]^-$	1.94	253.1809, 235.1716	$C_{21}H_{36}O_8$	Atractyloside G	Sesquiterpenes

16	14.22	219.1743	219.1749	$[M+H]^+$	-2.69	201.1639, 177.1273, 163.1117, 149.0962, 123.1168, 111.0805	$C_{15}H_{22}O$	(+)-Eudesma-4(14),7(11)-dien-8-one	Sesquiterpenes
17	20.94	230.1538	230.1545	$[M+H]^+$	-3.11	215.1307, 213.1273, 185.1328, 174.0914, 160.0759	$C_{15}H_{19}NO$	Atractylenolideamide	Sesquiterpenes
18	21.79	233.1534	233.1542	$[M+H]^+$	-3.24	215.1430, 205.1588, 197.1325, 187.1482	$C_{15}H_{20}O_2$	3β-Hydroxyatractylon	Sesquiterpenes
19	22.95	233.1535	233.1542	$[M+H]^+$	-2.81	215.1430, 187.1481	$C_{15}H_{20}O_2$	Costunolide	Sesquiterpenes
20	23.17	217.1585	217.1592	$[M+H]^+$	-3.41	199.1481, 175.1481, 161.0960, 159.1168	$C_{15}H_{20}O$	Atractylon	Sesquiterpenes
21	23.26	177.0545	177.0552	$[M+H]^+$	-3.78	149.0598, 145.0285	$C_{10}H_8O_3$	4-Methylumbelliferone	Coumarins
22	23.29	709.3288	709.3283	$[M-H]^-$	0.73	101.0607	$C_{32}H_{54}O_{17}$	Atractyloside H	Sesquiterpenes
23	23.32	233.1534	233.1542	$[M+H]^+$	-3.24	215.1430, 205.1586, 197.1327, 187.1481, 177.0910, 151.0754	$C_{15}H_{20}O_2$	Atractylenolide II	Sesquiterpenes
24	24.31	199.0752	199.0759	$[M+H]^+$	-3.54	181.0642, 171.0805, 153.0698, 143.0856, 128.0621, 115.0543	$C_{13}H_{10}O_2$	Atractylodinol	Polyacetylenes
25	24.93	183.0804	183.0810	$[M+H]^+$	-3.22	165.0699, 152.0620	$C_{13}H_{10}O$	Atractylodin	Polyacetylenes
26	25.14	249.1483	249.1491	$[M+H]^+$	-3.09	231.1379, 213.1274, 189.1275, 171.1166, 123.0804	$C_{15}H_{20}O_3$	Atractylenolide III	Sesquiterpenes
27	25.91	231.1379	231.1385	$[M+H]^+$	-2.62	213.1272, 185.1325, 157.1012, 143.0857	$C_{15}H_{18}O_2$	Atractylenolide I	Sesquiterpenes
28	26.14	709.3291	709.3283	$[M-H]^-$	1.16	101.0607	$C_{32}H_{54}O_{17}$	Atractyloside H isomer	Sesquiterpenes
29	27.41	205.1950	205.1956	$[M+H]^+$	-3.05	149.1326, 135.1169, 123.1168, 109.1012	$C_{15}H_{24}$	β-Farnesene	Sesquiterpenes
30	27.62	177.0546	177.0552	$[M+H]^+$	-3.22	149.0598, 145.0285	$C_{10}H_8O_3$	4-Methylumbelliferone isomer	Coumarins
31	27.62	219.1743	219.1749	$[M+H]^+$	-2.69	201.1639, 175.1481, 159.1170, 145.1013, 123.0805	$C_{15}H_{22}O$	Eudesma-4(15),7(11)-dien-8-one	Sesquiterpenes
32	41.29	279.2328	279.2324	$[M-H]^-$	1.41	261.2218, 243.2164	$C_{18}H_{32}O_2$	Linoleic acid	Organic acids

33	45.91	255.2329	255.2324	[M-H]$^-$	1.94	237.2243	$C_{16}H_{32}O_2$	Palmitic acid	Organic acids
34	47.23	281.2486	281.2481	[M-H]$^-$	1.94	263.2388	$C_{18}H_{34}O_2$	Oleic acid	Organic acids

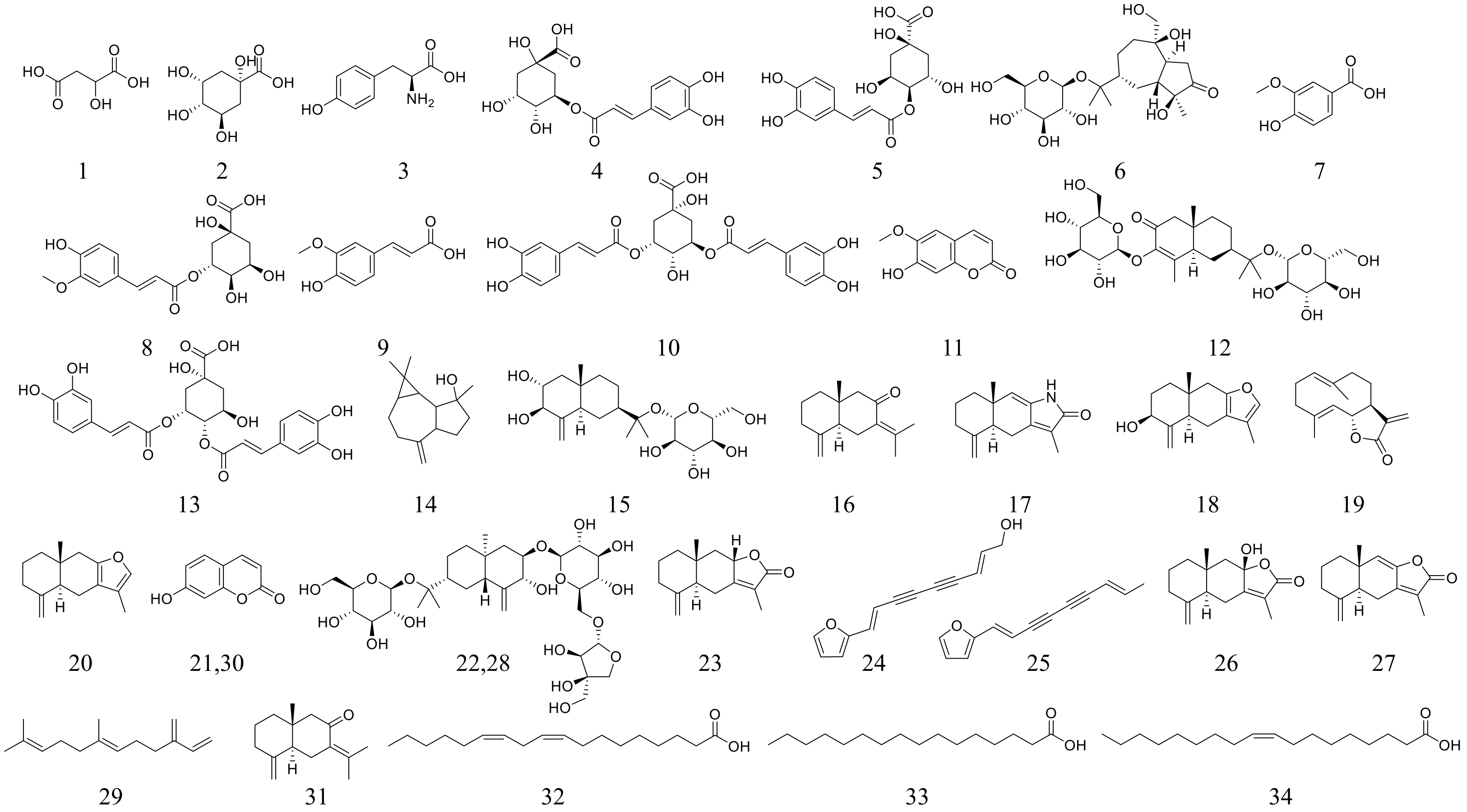

Figure S1. Identified chemical constituents in Atractylodis Rhizoma
